# Second generation androgen receptor antagonists and challenges in prostate cancer treatment

**DOI:** 10.1038/s41419-022-05084-1

**Published:** 2022-07-21

**Authors:** Yanhua Chen, Qianqian Zhou, William Hankey, Xiaosheng Fang, Fuwen Yuan

**Affiliations:** 1 Academy of Integrative Medicine, Shanghai University of Traditional Chinese Medicine, 201203 Shanghai, China; 2 Department of Genetics, The University of North Carolina at Chapel Hill, Chapel Hill, NC 27599 USA; 3 Department of Hematology, Shandong Provincial Hospital Affiliated to Shandong First Medical University, 271000 Jinan, Shandong China

**Keywords:** Cancer, Drug discovery

## Abstract

Prostate cancer is a hormone-dependent malignancy, whose onset and progression are closely related to the activity of the androgen receptor (AR) signaling pathway. Due to this critical role of AR signaling in driving prostate cancer, therapy targeting the AR pathway has been the mainstay strategy for metastatic prostate cancer treatment. The utility of these agents has expanded with the emergence of second-generation AR antagonists, which began with the approval of enzalutamide in 2012 by the United States Food and Drug Administration (FDA). Together with apalutamide and darolutamide, which were approved in 2018 and 2019, respectively, these agents have improved the survival of patients with prostate cancer, with applications for both androgen-dependent and castration-resistant disease. While patients receiving these drugs receive a benefit in the form of prolonged survival, they are not cured and ultimately progress to lethal neuroendocrine prostate cancer (NEPC). Here we summarize the current state of AR antagonist development and highlight the emerging challenges of their clinical application and the potential resistance mechanisms, which might be addressed by combination therapies or the development of novel AR-targeted therapies.

## Facts

- Second-generation AR antagonists including enzalutamide, darolutamide, and apalutamide for prostate cancer treatment increase patient survival.
- Second-generation AR antagonists only provide a temporary response and resistance eventually develops.
- Diverse mechanisms were reported regarding the resistance to second-generation AR antagonists.

## Open questions

- What is the mechanism of treatment-induced NEPC (t-NEPC) and what is the connection between t-NEPC and second-generation AR antagonists?
- How to address the second-generation AR antagonist-induced resistance?
- What is the progress of alternative AR-targeted therapy?

## Introduction

The incidence of prostate cancer ranks second among men worldwide and represents one of the leading causes of cancer death, with an estimated about 1.4 million new cases and 375,000 deaths worldwide in 2020 [1]. Prostate cancer onset and progression are closely correlated with the androgen receptor (AR) activity [2, 3] The activation of AR is mediated by androgens, whose synthesis is regulated by the hypothalamic–pituitary–testicular (HPT) axis [4]. As a result of the indispensable role of AR in prostate cancer, a number of anti-AR drugs have been developed and approved for different stages of prostate cancer in the past 30 years (Table 1). The first-generation AR antagonists included flutamide [5, 6], nilutamide [7, 8], and bicalutamide [9, 10], which were approved by the FDA in 1989, 1995, and 1996, respectively. While the patients respond to first-generation AR antagonists in the early stages of the disease, they eventually acquire resistance and progress to lethal stage castration-resistant prostate cancer (CRPC) [11]. Accumulating data indicate that restoration of AR signaling is critical for disease progression in these patients, as AR overexpression, especially due to AR genomic amplification, has been frequently observed and proven to be a principal driver of prostate cancer progression, both in clinical CRPC patients and in preclinical prostate cancer cell models [12–16]. The continued importance of the AR pathway in CRPC has encouraged researchers and clinicians to develop a second generation of AR antagonists with higher AR binding affinity and specificity to target aberrant AR signaling in lethal stage CRPC patients. Patient survival has indeed increased with the application of second-generation AR antagonists, which have higher AR binding affinity and inhibit AR more efficiently [17–22]. On the other hand, these agents have only provided a temporary response, due to the rapid development of resistance [23–25]. This review will discuss the development of AR antagonists, the limitations of current AR antagonists, and the mechanisms of resistance to these agents, and will outline emerging strategies to combat resistance and prolong patient survival.

**Table 1:** Timeline for the development of AR antagonists for prostate cancer.

Generic name	Other name	Approval date (or clinical stage)	Treatments
*Fist-generation*			
Flutamide	Eulexin	27 Jan 1989	mCRPC
Bicalutamide	Casodex	04 Oct 1995	mCRPC
Nilutamide	Nilandron	09 Sep 1996	mCRPC (combined with surgical castration)
*Second-generation*			
Enzalutamide	MDV3100	31 Aug 2012	mCRPC
		13 Jul 2018	nmCRPC
		16 Dec 2019	mCSPC
Apalutamide	ARN-509	14 Feb 2018	nmCRPC
		17 Sep 2019	mCSPC/mCRPC
Darolutamide	ODM-201	30 Jul 2019	nmCRPC
*Candidates in clinical trials*			
Proxalutamide	GT-0918	Phase II (recruiting)	mCRPC
BMS-641988		Phase I (closure)	CRPC
TQB3720		Phase I (recruiting)	mCRPC
SHR3680	Rezvilutamide	Phase I/IIA (complete)	mCRPC
TRC-253		Phase I/IIA (complete)	mCRPC

Information is taken from the websites ClinicalTrials.gov (https://clinicaltrials.gov/ct2/home) and Drugs@FDA: FDA-Approved Drugs (https://www.accessdata.fda.gov/scripts/cder/daf/).

## Development of second-generation AR antagonists

### FDA approved second-generation AR antagonists

**Fig. 1:**
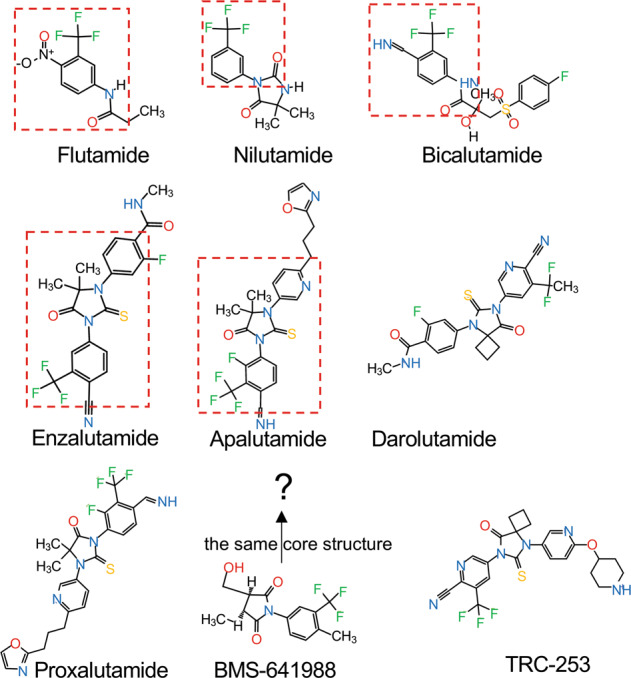
The 2D structure of AR antagonists. The red dotted box indicates the shared structure between drugs. Drug structure resources from PubChem (https://pubchem.ncbi.nlm.nih.gov/search/search.cgi).

**Fig. 2:**
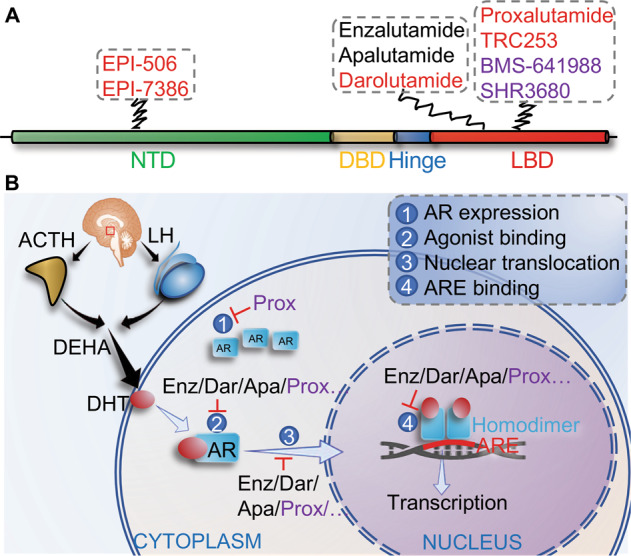
The binding location of AR antagonists and roles in inhibition of AR-mediated transactivation. **A** All of the FDA-approved second-generation AR antagonists bind to the ligand-binding domain (LBD). Potential AR antagonists including proxalutamide (Prox), TRC253, BMS-641988, and SHR3680 bind to the LBD, while EPI-506 and EPI-7386 bind to the N-terminal domain (NTD). Among these AR antagonists, EPI-506 and EPI-7386, darolutamide, proxalutamide, and TRC253 can bind with AR harboring mutations such as F876L. **B** All of the AR antagonists that bind to the LBD can competitively inhibit DHT binding to AR, as well as AR nuclear translocation and binding to DNA and coactivators. Binding of EPI-506 and EPI-7386 to the NTD of AR can inhibit AR transcriptional activation. Of note, proxalutamide can also repress AR protein expression.

Enzalutamide (also named MDV3100) is the first FDA-approved second-generation AR antagonist for the treatment of CRPC and exhibits a much higher AR-binding affinity in comparison to the first-generation AR antagonists. It competitively binds to the ligand-binding domain (LBD) of AR and inhibits androgen binding, nuclear translocation, DNA binding, and co-activator recruitment [26, 27]. Enzalutamide significantly prolongs the overall survival and metastatic-free survival of CRPC patients [17, 28, 29], and was approved by the United States FDA for treatment of metastatic CRPC (mCRPC) and non-metastatic CRPC (nmCRPC) in 2012 and 2018, respectively. Enzalutamide was also found to markedly prolong the castration-resistant free survival time of patients with castration-sensitive prostate cancer (CSPC) [18], and was approved for the treatment of CSPC in 2019. Although enzalutamide is widely used in clinical treatment for both CSPC and CRPC, the high steady-state brain level of enzalutamide has been found in clinical practice to be associated with central nervous system (CNS)-related events such as seizure, as it can antagonize the GABAα receptor in the CNS [29–32]. Another AR antagonist with a lower steady-state brain level subsequently emerged in the form of apalutamide (ARN-509), which shares the same core structure with enzalutamide (Fig. 1), but is associated with fewer seizure side effects [33]. Apalutamide is similarly considered to be a full AR antagonist, as it has high binding affinity with the LBD of AR [33]. Apalutamide can significantly increase the metastasis-free survival of nonmetastatic CRPC as well as the overall survival of metastatic CSPC [20, 34, 35], and was approved for nmCRPC in 2018 and for mCRPC/mCSPC in 2019. Both enzalutamide and apalutamide function as AR antagonists by inhibiting multiple stages of AR-mediated transcription, including by competing with DHT for AR binding, blocking AR nuclear translocation, and blocking DNA binding and cofactor recruitment [26, 33]. In contrast to enzalutamide and apalutamide, the most recently approved second-generation AR antagonist darolutamide (ODM-201) has a different chemical structure (Fig. 1) and cannot cross the brain–blood barrier [36], suggesting a lower potential for CNS side effects. Clinical trials have indicated that darolutamide provides not only meaningful antitumor effects but also a favorable safety profile [37, 38]. A randomized, double-blind, placebo-controlled, phase 3 trial involving men with nmCRPC has demonstrated significantly longer survival with darolutamide (40.4 months) than with placebo (18.4 months) [39]. A randomized, double-blind, placebo-controlled phase 3 study of darolutamide plus ADT versus placebo plus ADT in men is ongoing to assess the efficacy and safety of darolutamide in combination with standard ADT in metastatic hormone sensitive prostate cancer (mHSPC) patients (NCT04736199) (Fig. 2). Additional in vitro data have shown that darolutamide exhibits a consistent ability to efficiently inhibit full-length AR harboring a number of different characterized gain-of-function mutations [40]. Taken together, enzalutamide, apalutamide, and darolutamide are considered “pure” AR antagonists with the ability to suppress AR activation in CSPC, mCRPC, and nmCRPC patients [41]. Several concerns still persist, such as enzalutamide-induced seizures in CNS due to its high levels in brain, and the shorter serum half-life of darolutamide that has required higher doses and more frequent administration, leading to toxic effects such as cardiovascular disease [42, 43]. More importantly, prolonged use of AR antagonists induces drug resistance that rapidly attenuates their clinical benefits, motivating scientists to further explore new AR antagonists and alternative therapeutic strategies.

### Emerging AR antagonists in clinical trials

Several clinical trials are currently underway investigating novel AR antagonists with the potential to conquer the shortcomings of the present AR antagonists (Table 1). Proxalutamide (GT-0918) is a full AR antagonist which has 3-fold higher binding affinity in comparison with enzalutamide [44, 45]. It can also down-regulate the protein level of AR in CRPC. More importantly, luciferase reporter assays have shown that proxalutamide has the same effect on wild-type AR and on AR with clinically observed mutations (including F877L, W747C, and H875Y) that confer resistance to 1st or 2nd generation AR antagonists [44]. A phase I dose-escalation study of proxalutamide to evaluate its safety, pharmacokinetics, and antitumor efficacy in 16 patients with CRPC has shown a high degree of tolerance and promising antitumor activity in CRPC [44]. An open-label, randomized, expanded/phase II study is currently recruiting subjects with mCRPC who have progressed after either abiraterone or enzalutamide treatment in order to evaluate the safety and tolerability of proxalutamide and determine the dose level for phase III and/or other confirming studies (NCT03899467). BMS-641988 is another promising AR antagonist which was first reported by Salcati’s laboratory [46, 47]. BMS-641988 has comparable AR-binding affinity with proxalutamide and has shown an antitumor effect superior to bicalutamide in CWR22-BMSLD1 and LNCaP tumor xenograft models [46, 47]. In a phase I clinical trial to define the safety and tolerability of oral BMS-641988 in patients with CRPC, the therapeutic dose of BMS-641988 exhibited promising anti-tumor activity but was associated with an episode of seizure activity that led to study closure [48]. Although BMS-641988 did not progress further in clinical trials, it may be possible to design novel AR antagonists based on its core structure that have a reduced ability to concentrate in the brain, similar to the relationship between enzalutamide and apalutamide. While enzalutamide and apalutamide share the same core structure, the seizure side effect associated with enzalutamide is dramatically decreased with apalutamide because of its shorter half-life and lower steady-state level in the brain. Another novel AR antagonist, SHR3680, preclinically has shown anti-tumor potency comparable to enzalutamide but with a reduced distribution in the brain and significantly decreased risk to induce a seizure. A phase 1/2 study of patients with progressive mCRPC has shown that SHR3680 is well tolerated and safe, with promising anti-tumor activity across all doses tested in patients [49].

Agents that target regions of AR other than the LBD are in development as well, with the potential to counteract constitutively activating AR splice variants and AR point mutations. EPI-7386, for instance, is a second-generation NTD inhibitor (aniten) that is more active and more metabolically stable than EPI-506 (EPI-002 pro-drug) and has demonstrated a 20-fold improvement in AR-driven cellular potency compared to EPI-002 [50]. EPI-7386 inhibits cell proliferation across a panel of prostate cancer cell lines, including those driven by the AR variant AR-V7, can control tumor growth and induce tumor regression in several CRPC xenograft models, and is well tolerated in animal models [50, 51]. A phase 1 dose-escalation clinical trial of EPI-7386 in mCRPC patients is underway to assess its safety and to find a dose that can be given without unacceptable side effects (NCT04421222), as well as a phase 1/2 clinical trial of EPI-7386 in combination with enzalutamide in patients with mCRPC progressing on the standard of care therapies including second-generation anti-androgens (NCT05075577). TRC-253 is another novel AR antagonist that functions as a high-affinity competitive inhibitor of both wild-type AR and AR harboring mutations within the LBD [52]. A multi-center, first-in-human, open-label, Phase 1/2A dose-escalation study conducted in eligible mCRPC patients has indicated that high doses of TRC-253 are associated with some adverse events such as anemia (NCT02987829). It is not yet clear whether combination therapy of low dose of TRC-253 with other androgen inhibitors such as abiraterone can lower its toxicity and improve the anti-tumor effect. A more recent study has developed several AR antagonists based on the structure of darolutamide, from which “compound 28t” has been found to show superior efficacy against two resistant mutants (AR-F876L and AR-T877A) relative to darolutamide [53]. Further clinical trials are needed to assess its safety, pharmacokinetics, and efficiency. Additional candidates are anticipated as the next generation of AR antagonists emergent and enter clinical trials.

## Challenges of second-generation AR antagonists

### Off-target effects of AR antagonists

Although second-generation AR antagonists have become mainstays for the treatment of both CSPC and CRPC patients, their clinical benefits have been limited by potential side effects and especially by induced drug resistance. As mentioned above, the long half-lives and high levels of enzalutamide and BMS-641988 in the CNS may induce seizures in a small proportion of prostate cancer patients, as these AR antagonists can competitively bind and inhibit GABA-α activity [18, 32, 48, 54, 55]. A retrospective observational study has reported falls as a CNS-related event in patients with metastatic prostate cancer receiving enzalutamide (4.6%) [31]. Additionally, the clinical application of second-generation AR antagonists may increase the risk of cardiovascular events [56, 57]. A recent meta-analysis involving 4110 nmCRPC patients treated with enzalutamide, darolutamide, or apalutamide has indicated that the application of second-generation AR antagonists is associated with significantly increased risk of cardiovascular events including stroke, heart failure, and peripheral vascular disease [42]. This is consistent with previous enzalutamide studies, especially among prostate cancer patients with pre-existing cardiovascular disease, for whom enzalutamide may increase the risk of hypertension, likely driven by mineralocorticoid excess [58–61]. At the same time, the incidence of cardiovascular disease associated with second-generation AR antagonists is significantly decreased in comparison with other AR inhibitors such as abiraterone (CYP17 inhibitor) [62].

### AR antagonists-induced drug resistance and cancer evolution

Overall, administration of second-generation AR antagonists to patients in different stages of disease has resulted in a moderate survival benefit. However, several studies have indicated that about 30–60% of patients who receive second-generation AR antagonists eventually progress to death [17, 18, 20, 28, 29, 34, 39]. A proportion of these patients are primarily resistant to the treatment, which may be caused by AR heterogeneity in prostate cancer [63, 64] and other alterations in enzymes crucial for the conversion of extragonadal precursors to potent androgens, such as the 3βHSD1 germline variant [4]. In addition to de novo resistance, patients who receive second-generation AR antagonists inevitably develop acquired resistance within a variable period of time [65], which represents the greatest challenge of AR antagonists in prostate cancer treatment. Treatment-induced lethal NEPC (t-NEPC) progression is increased by the application of AR antagonists, especially in patients who have undergone enzalutamide treatment. De novo NEPC accounts for <2% of all prostate cancer at the time of diagnosis [66, 67], but the incidence of NEPC has significantly increased with the clinical application of AR inhibitors [68–71]. The current incidence of NEPC accounts for 18–20% of patients with CRPC, coinciding with the widespread clinical use of AR antagonists [68, 71].

## Emerging mechanisms of resistance to AR antagonists

### AR alterations and dysregulation in CRPC

**Fig. 3:**
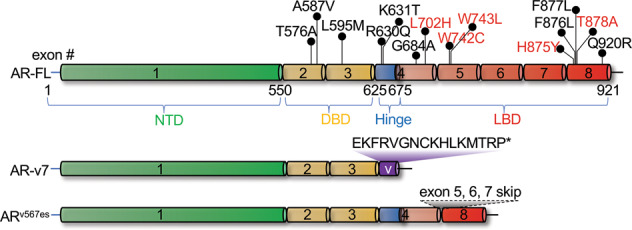
Recurrent AR mutations and alternative splicing variants contribute to AR antagonist resistance. Mutations in red are the most prevalent mutations in patients [74, 75], while those in black are enzalutamide- and apalutamide-resistant mutations [76, 77]. AR-v7 lacks exons 4/5/6/7/8 and differs by 16aa at the C-terminus compared with AR-FL. Exons 5/6/7 are excluded in AR^v567es^ compared with AR-FL.

Genomic analyses have indicated that 15–20% of CRPC patients harbor AR mutations [72, 73]. Collectively, over 150 mutations have been reported by the Androgen Receptor Gene Mutations Database within the LBD domain of AR in the context of prostate cancer, including single point mutations, pre-termination, deletions, and insertions. L702H, T878A, H875Y, W742C, and W743L are the most prevalent mutations reported in clinical prostate cancer patients (Fig. 3) [74, 75]. These point mutations in the LBD may result in lower AR antagonist binding affinity or even conversion of the AR antagonist into an agonist. Studies have demonstrated that multiple point mutations confer resistance to enzalutamide and apalutamide, including A587V, F876L, F877L, G684A, K631T, L595M, Q920R, R630Q, T576A, and T878A [76, 77]. F876L in particular triggers an antagonist-to-agonist switch that drives phenotypic resistance to enzalutamide [76]. Additionally, enzalutamide can act as a weak partial agonist in CRPC patients who harbor F877L, and becomes a strong agonist in patients harboring both F877L and T878A mutations [78]. Such mutations do not appear to be prevalent among clinical prostate cancer patients, and although anti-androgen withdrawal syndrome (AAWS) frequently occurs after discontinuation of first-generation anti-androgen therapy, but it is rarely observed in enzalutamide treated patients [79–81].

AR alternative splicing (AS) events resulting in the absence of the LBD from AR isoforms is another major resistance mechanism relevant to prostate cancer [82]. The human *AR* gene is comprised of eight canonical exons (Fig. 3) encoding the NTD, DNA-binding domain (DBD), hinge region, and LBD [83]. At least 18 splicing variants have been reported, as summarized by Lu et al. [84] and Snow et al. [85], most of which do not contain the LBD targeted by second-generation AR antagonists. Importantly, AR variants such as AR-v7 and AR^v567es^ arise from intricate *AR* genomic arrangements and dysregulation of AS factors [63, 64], and have been shown to be constitutively active in CRPC patients and prostate cancer cell models [86–89]. AR-v7 and AR^v567es^ can induce the expression of genes that regulate cell growth and survival independently of their interaction with full-length AR (AR-FL) [90–92]. On the other hand, constitutively active AR-v7 and AR^v567es^ also promote the function of AR-FL by facilitating its nuclear localization and DNA binding in the absence of androgen or even in the presence of enzalutamide [90, 91, 93, 94].

AR overexpression is also considered a mechanism of resistance to AR antagonists, and can result from *AR* gene amplification, enhanced transcription activation, or increased AR stability at the mRNA/protein level. Studies have shown that about 80% of tumors overexpressing AR exhibit *AR* amplification [95, 96]. Systematic analysis across different CRPC patients has shown that ~30% have amplification of the *AR* locus, and that AR overexpression is sufficient to confer resistance to AR antagonists in clinical practice [65, 74, 97]. Notably, AR amplification is more common in patients who have progressed on enzalutamide compared to abiraterone or other agents (53% vs. 17% or 21%) [98]. Enhanced *AR* transcription or increased stability of AR protein/mRNA is sufficient to upregulate AR levels without *AR* gene amplification [99–101], and can facilitate tumor growth despite minimal androgen [102]. Interestingly, a recent study has found that MYB interaction with AR can sustain its ligand-independent activation and promote castration resistance in prostate cancer [101].

### Reprogramming of AR transcriptional activity by AR antagonists

Although second-generation AR antagonists are typically considered “pure” antagonists, our recent studies have found that these agents function as partial agonists as well [103, 104]. We have demonstrated that in the presence of enzalutamide or darolutamide, AR is enriched in the distal elements of cancer-related genes such as *NR3C1* (encoding GR) and *SLC7A11*, and upregulates their expression in both ADPC and CPRC cell models. Transcriptome analysis further demonstrates that enzalutamide induces global upregulation of a number of cancer-related genes. Mechanistic studies have revealed that this process is assisted by the pioneer factor GATA2 and the mediator complex [104–107]. Interestingly, comparative analysis of the cistrome and transcriptome profiles of AR and GR has shown a high degree overlap [108, 109]. These results are consistent with earlier findings from Sawyer’s lab that GR upregulation contributes to enzalutamide and apalutamide resistance in LNCaP and VCaP cell models [12, 108]. ChIP-seq studies have revealed that agonist-liganded AR and antagonist-liganded AR bind to two different motifs, leading to distinct transcriptional outcomes in prostate cancer cells [103]. In conclusion, second-generation agents previously thought to function as pure AR antagonists might also perform a partial agonist function that reprograms AR transcriptional activity to transcribe oncogenes that counteract their AR antagonist role. Targeting GATA2, which mediates the agonist role of enzalutamide, with a small molecular inhibitor can re-sensitize both ADPC and CRPC cell models to enzalutamide treatment [104]. This indicates that prostate cancer treatment may benefit from combining AR antagonist therapy with inhibitors targeting the AR co-factors that facilitate antagonist-induced reprogramming of AR transcriptional activity. Further experiments are necessary to identify the most critical AR antagonist-specific co-factors.

### Heterogenetic evaluation independent of AR

Tumor heterogeneity is one of the major drivers of cancer progression and represents one of the primary challenges in cancer treatment. Tumor heterogeneity exists both in nascent prostate cancers and following antagonist-driven evolution, and contributes to CRPC progression and drug resistance [110–114]. As described above, heterogeneity within the *AR* locus alone can range from AR LBD point mutations to alternative splicing events, to overexpression that increases the sensitivity of AR to hormone stimulation, to loss of the antagonist binding region or other changes that mediate antagonist-agonist switching of the second-generation AR-targeted therapies [63, 64]. Outside of its impacts on the *AR* gene, tumor heterogeneity can also contribute to AR antagonist resistance through other pathways independent of AR.

Lineage plasticity is driven by alterations in *PTEN*, *RB1*, *TP53,* or *SOX2* enables tumors to become AR independent and activates neuroendocrine differentiation, which is emerging as an increasingly recognized mechanism of resistance to AR-targeted therapies. It has not been established whether these alternations pre-exist within a subset of prostate epithelium cells that are intrinsically resistant to AR-targeted therapies or whether they are induced during the course of treatment with AR-targeted therapies. A recent whole-exome sequencing and immunohistochemistry (IHC) study in 37 prostate cancer patients before ADT has shown that loss of chromosome 10q (containing *PTEN*) and alterations to *TP53* are predictive of poor response to enzalutamide. A subset of prostate cancer exhibits greater histologic and genomic diversity, accompanied by a higher fitness to resist therapy [112]. In addition to the pre-existing heterogeneity of the tumor, the application of second-generation AR antagonists is associated with treatment-induced heterogenetic evolution [115]. A related fact is that the increasing use of AR antagonists such as enzalutamide in CRPC settings has favored the increase in incidence of t-NEPC [70, 115]. Recurrent amplification or loss of function of genes such as *PTEN, RB1*, and *TP53* are characteristic of treatment-induced NEPC patients [116–118]. Experiments in cellular models also demonstrate that overexpression of *MYC* or knockdown of *PTEN/RB1/TP53* drives lineage plasticity models to shift from CRPC to NEPC [119–122].

**Fig. 4:**
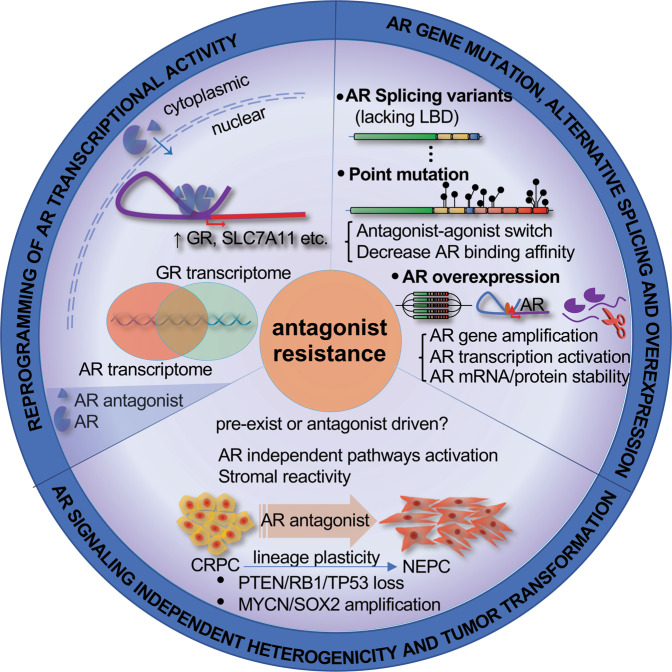
Mechanisms of resistance to androgen receptor inhibitors in prostate cancer. The partial agonist role of second-generation AR antagonists induces the expression of cancer-related genes including GR. GR in turn regulates the expression of a set of genes that overlaps with AR downstream pathways. AR alterations can include alternative splicing, point mutation and overexpression. Other AR signaling-independent mechanisms such as PTEN/TP53/RB1 loss of function and MYCN/SOC2 activation can mediate CRPC progression and contribute to AR antagonist resistance in CRPC.

Activation or deactivation of other pathways including Wnt-β-catenin, PI3K–AKT-mTOR, and DNA repair are also reported to be associated with resistance to AR antagonists, as summarized by Schmidt et al. [24, 123]. Stromal reactivity (SR) surrounding tumors can also shape the dynamics of prostate cancer evolution and tumor aggressiveness [124]. Further studies are needed to determine the extent to which AR antagonists can drive the cross-talk of intricate intercellular signaling networks between the tumor and stromal cells. Although the source of tumor heterogeneity in CRPC remains unclear, it is increasingly recognized that this phenomenon contributes to second-generation AR antagonist resistance and NEPC progression. In summary, the evolution of CRPC resistance and progression results from the combined contributions of both AR-dependent and AR-independent pathways (Fig. 4).

## Strategies to overcome resistance to AR Antagonists

### Development of novel AR-targeted therapies

In reviewing the mechanisms of AR antagonist resistance, changes in the AR signaling pathway stand out as one of the primary reasons, with accumulating studies demonstrating that events including AR point mutation, rearrangement, amplification, and transcriptional upregulation of AR variants can result in the failure of AR antagonist treatment and in some cases can mediate antagonist–agonist switching. Additionally, side effects such as seizure and cardiovascular disease have limited the clinical benefits of second-generation AR antagonists. Although darolutamide and proxalutamide show clinical efficacy in CRPC patients harboring point mutations within the AR LBD [40, 44], these antagonists cannot target AR variants such as AR-v7 or AR^v567es^ that lack the LBD and contribute to prostate cancer progression and resistance to AR antagonists [93, 125–128]. As a result, drug development strategies are increasingly recognizing the need to screen for novel agents that can target both AR-FL harboring clinical mutations and AR variants that lack the LBD [129, 130]. As a result, AR-targeted therapies focusing on the N-terminal domain or DBD of AR have become a subject of intense interest as a potentially promising strategy to overcome AR heterogeneity in prostate cancer. Several N-terminal inhibitors and DBD inhibitors, such as EPI-7386 and SBF-1 have been reported in preclinical studies with promising ability to overcome many known mechanisms of resistance to existing hormonal therapies [131–134]. The highly selective N-terminal domain inhibitor EPI-7386 is currently in phase 1 and phase 1/2 clinical trials to evaluate the safety, tolerability, and preliminary efficacy of EPI-7386 alone and in combination with enzalutamide in mCRPC (NCT05075577 and NCT04421222). Another recent study has found that the well-characterized antitumor agent SBF-1 can selectively bind to the AR-DBD and block the transcription of AR target genes, and has been proven to repress prostate cancer growth both in vitro and in vivo [134]. Beyond targeting alternative AR domains, another strategy to overcome resistance conferred by point mutations in the LBD is the structure-based design of novel AR antagonists to specifically disrupt LBD dimerization, as AR transactivation potential requires LBD-mediated homodimer formation, regardless of the presence or absence of LBD point mutations [135].

The emergence of gene-targeted therapies for different diseases may be a more straightforward approach to confronting AR splicing variants and point mutations. PROteolysis-TArgeting Chimeras (PROTACs) have been recognized as a promising technology to chemically knock down targeted genes at the protein level, particularly in the context of cancer [136]. Preliminary clinical data on PROTAC ARV-110, which flags AR for degradation, have shown safety and efficacy in men with mCRPC (NCT03888612) [137, 138]. Gene knockdown technologies such as CRISPR-Cas9 directed gene deletion have shown promise but have not yet found clinical applications, as CRISPR-Cas9 directed DNA editing may cause unreversible and unpredictable mutations in chromatin in vivo and in vitro [139–141]. Although the approval of the first siRNA drug by the US FDA in 2018 marks the beginning of a new era of RNAi therapeutics, numerous studies demonstrated widespread off-target effects of siRNA-mediated gene silencing have similarly limited its clinical implementation [142–144]. The more recently-reported CRISPR-Cas13 system might circumvent these limitations, as it has been proven to target RNA with high specificity and efficiency [145, 146], and several studies have demonstrated that targeting driver oncogenes using CRISPR-Cas13 can repress the growth of different types of cancer both in vitro and in vivo [147–149]. However, further investigation is needed to evaluate the specificity and efficiency of CRISPR-Cas13 for AR targeted therapy specifically in the context of prostate cancer. in summary, accumulating studies indicate that the development of novel AR antagonists recognizing the NTD or LBD of AR and the gene-targeted therapies hold great promise to overcome the shortcomings of current AR antagonists (Table 2).

**Table 2:** Novel AR targeted therapies.

Agents/technologies	Mechanisms and preclinical/clinical evidence
AR DBD inhibitors	AR binding to the DNA via its DBD is an essential step in the regulation of gene transcription by both full-length and variant forms of AR [163]. AR DBD inhibitors can effectively inhibit the activity of truncated ARVs and repress PCa growth in vitro and in vivo [129, 134, 164].
AR NTD inhibitors	The AR NTD is essential for AR transactivation, and NTD deletion renders AR transcriptionally inactive [165]. A phase I trial has established the safety of EPI-506 and provides proof of concept for targeting the AR NTD [133].
AR-targeted PROTACs	PROTACs technology has emerged as a promising approach for targeted therapy in various diseases, particularly in cancer [136]. ARV-110 targets AR and is safe and has efficacy in mCRPC patients [137, 138]. A phase I/II dose escalation study is currently recruiting mCRPC patients to assess the tolerability and safety of ARV-110 (NCT03888612).
AR-targeted CRISPR-Cas13	CRISPR/Cas13 targeting of oncogenes has been proven to repress the growth of multiple types of cancer in vitro and in vivo [147–149].

### Combined therapies with AR antagonists

Although second-generation AR antagonists have prolonged prostate cancer survival time, side effects and the rapid evolution of drug resistance remain stumbling blocks associated with the use of AR antagonists in clinical practice. One possible contributor to both the side effects and the induction of drug resistance is the high dose of AR antagonists currently administered to patients. Indeed, our study has found that 25 μM of enzalutamide that imitates the real dose of enzalutamide in patients induced higher expression of cancer-related genes such as *GR* and *SLC7A11*, in comparison with 10 μM of enzalutamide [104]. Therefore, multipoint targeting of the AR signaling pathway may accomplish the same or even greater antitumor effect while reducing side-effects, which may slow down the induction of resistance and cancer progression. A meta-analysis of two phase 3 trials has shown that abiraterone and prednisolone, which target androgen synthesis, can combine with enzalutamide to significantly improve metastasis-free survival in high-risk non-metastatic prostate cancer [150]. A phase IB/IIA study of the pan-BET inhibitor ZEN-3694 in combination with enzalutamide showed acceptable tolerability and potential efficacy in patients with androgen-signaling inhibitor-resistant mCRPC [151]. Other promising combined therapies involving immunotherapy, CDK inhibitors and radiotherapy are summarized in Table 3. Notably, we and other groups have demonstrated that enzalutamide and darolutamide can induce the expression of ferroptosis-related genes in both ADPC and CRPC [104, 152], which have proven to be correlated with prostate cancer recurrence [153, 154]. Targeting ferroptosis might be a novel therapeutic strategy for advanced prostate cancer, as ferroptosis inducers significantly decrease prostate cancer cell growth and migration in vitro and delay tumor growth of treatment-resistant prostate cancer in vivo, with no measurable side effects [155, 156]. Further clinical trials are needed to test the potential of this therapeutic strategy.

**Table 3:** Potential therapeutic combinations of AR antagonists with other agents.

Combined strategy	Examples	Preclinical or clinical evidence
AR antagonist+ Immunotherapy	Enza & CART cell (EPhA2)	Enza-induced EPhA2R expression in prostate cancer cells, as well as the ability of agonistic dimeric synthetic (135H12) and natural EPhA2R ligands to degrade EPhA2R and delay tumor migration and growth in mouse model [166].
AR antagonist+ AR cofactor inhibitor	Enza/Daro & GATA2/HSP90 inhibitor etc.	Enza/Daro combination with GATA2 inhibitor (K7174) inhibits PCa cell growth more effectively than Enza alone [104]. Co-targeting AR and HSP90 suppresses both PCa cell growth and Enza resistance. Bruceantin targeting of HSP90 overcomes resistance to hormone therapy in CRPC [167, 168].
AR pathway inhibitor (sequencing)	Abiraterone acetate followed by Enza	A multicenter, randomized, open-label, phase II, crossover trial has shown that a sequencing strategy of abiraterone acetate treatment followed by Enza provides a greater clinical benefit than the opposite treatment sequence [157].
AR antagonist+ AR independent target inhibitor	Enza & AU-15330 (PROTAC targets SWI/SNF)	AU-15330 induces potent inhibition of tumor growth in xenograft models and synergizes with Enza, even inducing disease remission in CRPC models without toxicity [169].
	Enza & Olaparib/Rucaparib (PARP inhibitor)	A RAMP phase Ib trial of rucaparib and Enza has shown safety and early efficacy [170]. Several clinical trials are underway to evaluate the potential of combinatorial therapy for mCRPC patients (NCT04455750/NCT03395197).
	AR antagonists & CDK4/6 inhibitor (e.g. palbociclib, abemaciclib)	The Cyclin-CDK-RB axis is critical to resistance to AR antagonists, and CDK inhibitors effectively inhibit cancer growth in vitro and in vivo [120, 171]. Clinical trials are underway to evaluate the combination of CDK-inhibitors with enzalutamide in CRPC patients (NCT03685591/NCT02555189).
AR antagonist+ radiotherapy	Enza & Stereotactic body radiotherapy/radium-223?	A study by Maughan et al. has shown the combination of Enza and radium-223 to be safe and associated with promising efficacy in men with mCRPC [172], while another group found limited benefit [173–175]. Metastasis-directed therapy (MDT) in mCRPC oligo-progressive lesions extends the efficacy of treatment with AR-targeted agents [176].

Sequencing treatment strategies with different inhibitors have achieved initial success in CRPC. A phase II clinic trial has shown that using a sequencing strategy of abiraterone acetate followed by enzalutamide in CRPC patients provides a clinical benefit [157], although further exploration is needed to determine whether these findings apply to patients who have previously received one of the androgen-directed agents in a hormone-sensitive setting [158, 159]. Adaptive therapy to cycle drug selection using real-time data to limit the length of exposure to one selective pressure should also be considered [160]. Although no trials of adaptive therapy to reduce resistance to second-generation AR antagonists are currently underway, a pilot study to assess adaptive abiraterone monotherapy has supported the potential of the adaptive therapy approach with AR antagonists (Table 3) [161].

Notably, although GR pathway activation has been considered one of the principal mechanisms of resistance to AR antagonists [23, 24, 41], a phase I/II clinical trial for enzalutamide and the GR antagonist mifepristone in mCRPC (NCT02012296) has shown that the combined treatment is safe and well tolerated, but does not delay time to PSA, radiographic or clinical PSA progression-free survival [162]. These preliminary results indicate that the development of more specific GR antagonists should be explored in combination with AR antagonists.

## Conclusions and perspectives

In conclusion, three second-generation AR antagonists have been developed through interdisciplinary efforts during the past decade, and their approval for prostate cancer treatment has significantly improved survival and decreased prostate cancer-related death worldwide, particularly in patients with mCSPC and CRPC. At the same time, drawbacks of these AR antagonists have gradually emerged, especially the ability of second-generation AR antagonists to induce resistance and progression of patients from CRPC to t-NEPC. Mechanistic studies indicate that *AR* alteration, reprogramming of AR transcriptional activity by induced AR antagonists, and both pre-existing and therapy-driven tumor heterogeneity contribute to prostate cancer resistance and tumor progression. These obstacles might be addressable through the joint efforts of both clinical doctors and basic researchers to develop novel inhibitors or other technologies targeting AR and to explore combination/sequencing therapeutic strategies.

## Data Availability

All relevant data are included in this manuscript.
